# Laser driven quantum rings: one byte logic gate implementation

**DOI:** 10.1039/c7ra11528h

**Published:** 2018-01-17

**Authors:** Dario Cricchio, Emilio Fiordilino

**Affiliations:** Dipartimento di Fisica e Chimica, Università di Palermo Via Archirafi 36 90123 Palermo Italy dario.cricchio@unipa.it emilio.fiordilino@unipa.it

## Abstract

We study the effect of the carrier-envelope-phase (CEP) on the high harmonic generation (HHG) from a quantum ring driven by two short orthogonal lasers polarized along the *x* and *y* axes. In particular, by varying only the phase of the laser polarized along *y* it is possible to control the intensity of the emitted harmonics. In fact, we show that the system can efficiently emit harmonics if the laser polarized along *y* is small and that the cut-off of the spectra can be controlled by changing the phase or the intensity ratio between the two lasers. The wavelet analysis of the emitted harmonics and the time dependence of the angular momentum and of the energy acquired by the electron show that the electron has several main angular momentum variations that generate the cut-off harmonics in as many pulses. These results may have a significant technological impact in computer technology to store information. The implementation of a logical gate that exploits the different temporal locations of the pulses is discussed.

## Abstract 2

We study the effect of the carrier-envelope-phase (CEP) on the high harmonic generation (HHG) from a quantum ring driven by two short orthogonal lasers polarized along the *x* and *y* axes.

## Graphical abstract

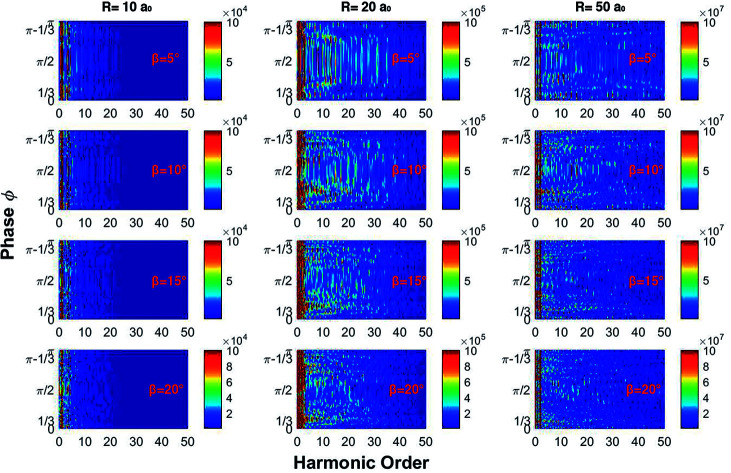

## Introduction

Experimental evidence demonstrates that atoms and molecules driven by an intense laser pulse of angular frequency *ω*_L_ irradiate new electromagnetic waves whose spectrum essentially contains harmonics of *ω*_L_. The intensity of the lines rapidly decreases for the first few harmonics, is almost constant along a wide plateau and then quenches in a quick cut-off. As a rule, scatterers with spherical symmetry emit only odd harmonics. The phenomenon, called high harmonic generation (HHG), provides an important benchmark to non-linear theories and seems to be on the verge of becoming an effective source of high frequency and coherent radiation; actual experiments show that the plateau can be as large as *ω*_M_ ≈ 100*ω*_L_^1–4^*i.e.* using a laser wavelength of *λ*_L_ = 800 nm = *ℏω*_L_ ≅ 1.55 eV (as in this paper), and that obtaining a cut-off energy of *ℏω*_M_ ≈ 150 eV is possible.

For applications as well as fundamental reasons, the control of the characteristics of the spectrum and, overall, of the extension of the plateau is of paramount interest and several ways of reaching these goals have been suggested. For instance, the most efficient coupling between radiation and matter is achieved when the laser frequency is resonant with a molecular transition, in this case, for large accelerations of the charges obtained even at intermediate laser intensity; thus the exploitation of resonances has been indicated as a viable solution.^5–12^

Also, large molecules and mesoscopic systems such as graphene and fullerene have large electric polarizability and can be effective sources of harmonics.^13–17^ A noteworthy system is the quantum ring (QR), a slender planar structure with a circular geometry. It presents several optical properties, in particular, when driven by a circularly polarized laser field, it emits a harmonic spectrum more intense than that emitted from benzene.^18^ Moreover, when driven by a two color laser field it can efficiently emit a broad harmonic spectrum.^19,20^ Concentric double quantum rings were used to investigate the Aharonov–Bohm oscillation. This oscillation was utilized to design logic gates with quantum rings.^21^

A mesoscopic ring treated with a magnetic flux is used to make NAND gates;^22^ in the experiment the ring is attached symmetrically to two metallic electrodes and two gate voltages are applied in one arm of the ring, these are treated as the two inputs of the NAND gate. Optical signals are used in ref. 23 where two silicon microring resonators driven by a monochromatic laser are used to perform XOR and XNOR operations. They use two electrical signals to control the resonant states of the microrings. By changing the states it is possible to control the logic output in order to have XOR and XNOR operations. In ref. 24 and 25 the authors create direct OR/NOR and AND/NAND logic circuits using two parallel microrings. In ref. 26 the possibility of generating optical NOT and XOR logic operations based on a terahertz optical asymmetric demultiplexer is shown.

In the theoretical description of a laser pulse with a duration of a few optical cycles, an important parameter is the so called carrier-envelope phase (CEP), which is the phase of the carrier wave with respect to the maximum of the envelope. Recently, its actual value has been shown to be an important tool for controlling the properties of the radiation.^27^

In this paper we study the emission of high harmonics using a QR driven by two orthogonal lasers polarized along the *x* and *y* axes. In particular, we are interested in the comprehension of the role of the CEP in the extension of the plateau and in the relative intensity of the harmonic lines in order to use the emitted harmonics as logic signals. To do this, we need to understand when the harmonics near the cut-off are emitted. Thus we perform a wavelet analysis of the spectra to study the time evolution of the intensity of the harmonics and show that the lines in the cut-off region are generated in several pulses with a 0.5 optical cycle (oc) duration; oscillations of the intensity of the harmonic lines with a frequency double that of the laser have been reported in similar calculations.^28,29^ These results and the presence of a residual angular momentum of the electron led us to suggest using a QR for storing information and for fabricating two/three/four-state logic gates. The all-optical system introduced here has the advantage that it exploits a process, such as harmonic generation, of the electronic systems that takes approximately 10^−15^ seconds instead of approximately 10^−7^ seconds. This is a great advantage because it speeds up all the logic operations by orders of magnitude. Furthermore, the absence of electrical circuits reduces power consumption due to the lack of dissipation in the form of heat and allows further miniaturization of the devices.

## Theory

To gain insight on the physics of the system under consideration we adopt a simplified model that does not shade the effects and helps with visualization. Thus we consider one electron bound to a one-dimensional ring of radius *R* lying in the *x*–*y* plane, driven by a laser field polarized in the same plane; no ionization is possible within the assumed model, and the three-step mechanism cannot be invoked to explain harmonic generation. We are interested in the range *λ*_L_ ≫ *R* thus the electric field may be written in the so called dipole approximation:1
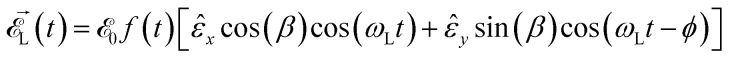
where ℰ_0_ is the electric field strength, *ε̂*_*x*_ and *ε̂*_*y*_ are unit vectors along the *x* and the *y* axes, respectively, and *f*(*t*) is the shape of the two laser pulses. The phase *ϕ* in the *y* component of the field denotes the carrier-envelope-phase (CEP) and is an important parameter in our subsequent investigation. The parameter *β* sets the relative strength of the two lasers; for *β* = 0° we have only the electric field along the *x* axis, for *β* = 45° the two electric fields have the same strength and, finally, for *β* = 90° we have only the second electric field. Finally, we remark that a correct description of a pulse profile requires that^4^2
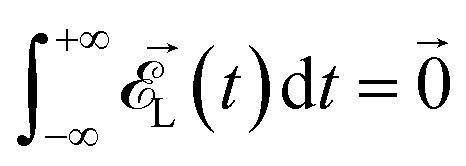
be approximately fulfilled with the set of parameters used in this paper for any value of *ϕ*.

The Hamiltonian of our system written in the length gauge is:3
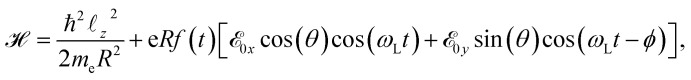
where *m*_e_ is the electron mass, *θ* is the angle between the electron position and the *x* axis, ℰ_0*x*_ = ℰ_0_ cos(*β*), ℰ_0*y*_ = ℰ_0*y*_ = ℰ_0_ sin(*β*), and 
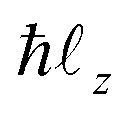
 is the *z* component of the angular momentum operator. The full wave function |*ψ*(*t*)〉 of the electron is a solution of the time dependent Schrödinger equation (TDSE)4
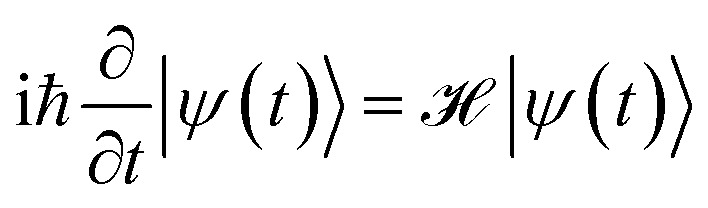
and can be expanded as5
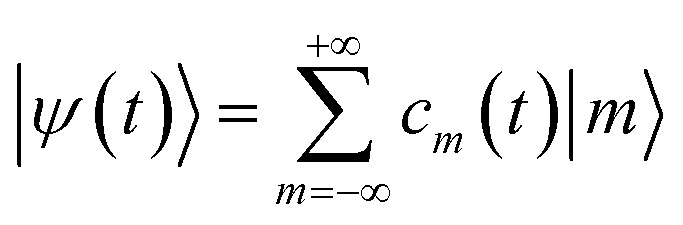
where 
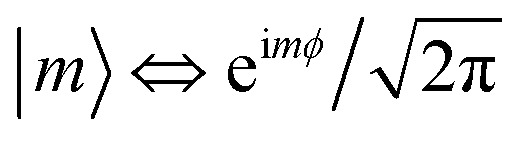
 are eigenstates of the laser-free Hamiltonian with energy *ℏω*_m_ = *ℏ*^2^*m*^2^/2*m*_e_*R*^2^ and orbital angular momentum *L*_*z*_|*m*〉 = *mℏ*|*m*〉. Substitution of |*ψ*(*t*)〉 into the TDSE leads to an infinite set of coupled differential equations for the probability amplitudes *c*_m_(*t*)6
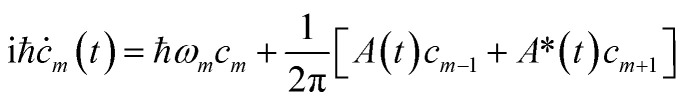
with *A*(*t*) = *ℏω*_L_*f*(*t*)[cos(*ω*_L_*t*) + i cos(*ω*_L_*t* − *ϕ*)] and with an initial condition *c*_*m*_(0) = *δ*_*m*,0_.

By defining 
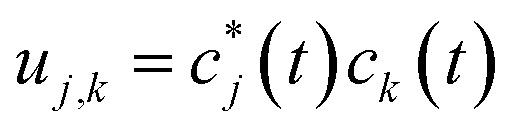
 we find the expectation value of the components of the charge position:7
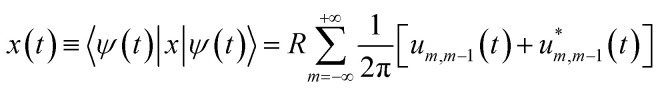
8
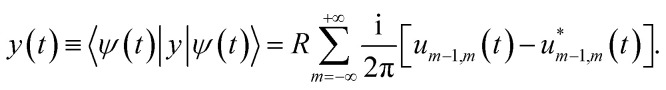


By differentiating two times *x*(*t*) and *y*(*t*) and with the help of the TDSE we find the electron acceleration *a⃑*(*t*) = (*a*_*x*_(*t*),*a*_*y*_(*t*)); and, using the classical Larmor formula:9
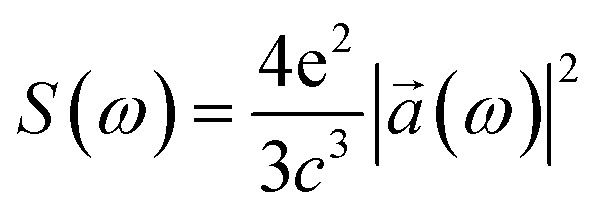
where *a⃑*(*ω*) = (*a*_*x*_(*ω*),*a*_*y*_(*ω*)) is the Fourier transform of *a⃑*(*t*), we find the energy irradiated *S*(*ω*)d*ω* in the frequency range [*ω*, *ω* + d*ω*] by the accelerating charge during the whole laser pulse. From the state |*ψ*(*t*)〉 we calculate the energy *K* and the angular momentum *L*_*z*_ acquired by the electron:10
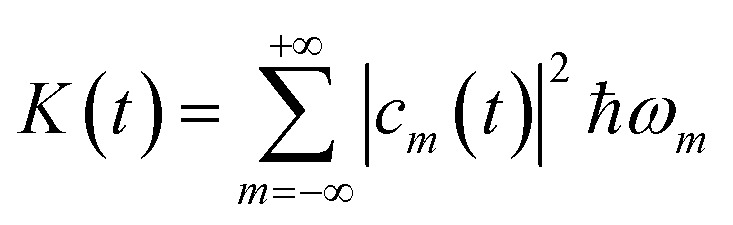
11
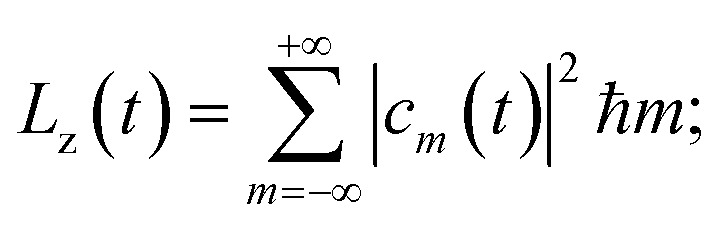
they are important quantities in the perspective of using the QR for storing information.

## Numerical simulations

In our calculations we have numerically solved the system of coupled differential eqn (6) from *m* = −10 to *m* = 10, and we use a QR with radii *R* = 10, 20, and 50 *a*_0_ (the Bohr radius), two orthogonal laser fields on the *x*–*y* plane with *λ*_L_ = 800 nm, a total intensity of 3 × 10^14^ W cm^−2^, *β* ∈ [5°, 20°], and a trapezoidal shape *f*(*t*) with 4 optical cycles (oc) of full duration and 0.5 cycles of on-off switching. We vary the phase *ϕ* between 0 and π with a step of 0.01π, obtaining 315 spectra (0, 0.01, …, 3.14) for each value of *β*. For the QR we have performed simulations for a large range of the radius *R* but here, for simplicity’s sake, we only discuss the case of *R* = 20*a*_0_.

**Fig. 1:**
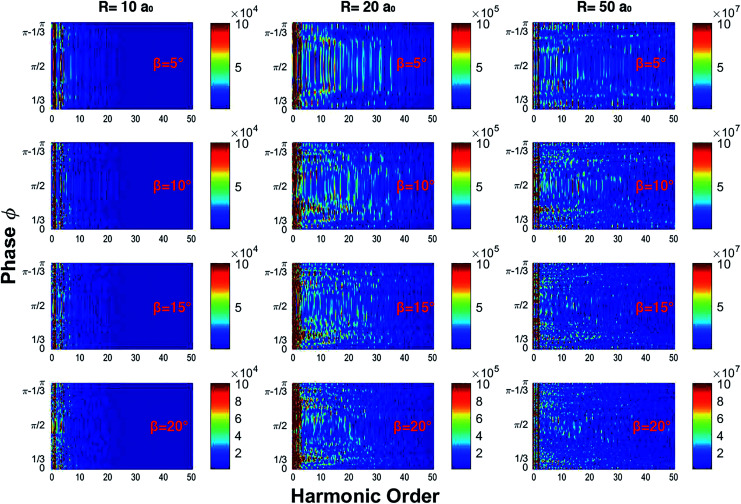
Harmonic spectra as a function of the phase *ϕ* for different values of the radius and *β*. By increasing *β* the harmonics split into several branches; this effect is more evident for *R* = 20*a*_0_.

In Fig. 1 we show the harmonic spectrum as a function of the calculated only phase *ϕ* and for *β* = 5°, 10°, 15°, and 20° in a color rendering scale. In the simulations we have seen that large radii correspond to a wide plateau of harmonics. At *R* = 20*a*_0_ the more intense harmonics (appearing in red in the rendering scale) extend up to the 20th harmonic order while the cut-off extends up to the 40th harmonic order. From the spectra, we notice that the first harmonic is almost independent from the phase *ϕ*. Finally, when the intensity of the component of the laser field along *y* grows beyond *β* = 30°, we do not observe phase dependent harmonic lines. We can affirm that the modulation of the harmonic lines as a function of the phase *ϕ* occurs only when the strength of the field polarized along the *y* axis is less than 30% of the full strength. In particular, we observe that for *β* ≤ 50° more intense harmonic lines are preferentially emitted for *ϕ* = π/2, but when increasing the *β* parameter they split into branches, which tend to *ϕ* = 1/3 and to *ϕ* = π − 1/3. The state of the art of the CEP lasers presents a CEP stability of sub-100 mrad.^30,31^ The most noticeable results of our calculations have a resolution of about 200–300 mrad, so we can say that we are well within today’s state of the art. In general, the short duration of the driving field causes broadening of the harmonic lines and the presence of noise in the spectra. However, for the particular problem dealt with now, deeper insight can be gained by studying the classical counterpart of the laser driven ring: it has been shown that the Newton equation of motion of a charge bound on a circle has a chaotic nature and this fact produces a signature in quantal calculations in the form of line broadening and noise.^32,33^

**Fig. 2:**
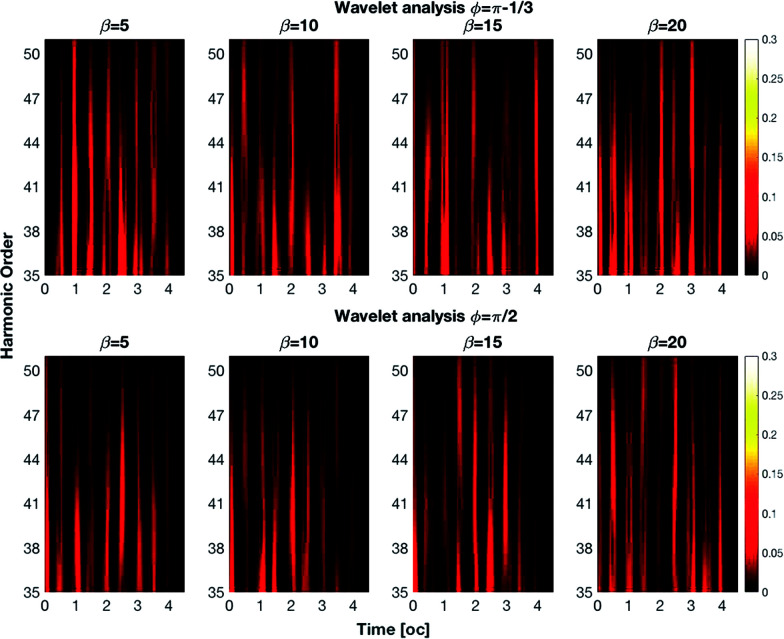
Wavelet analysis of the spectra for different values of *β* in degrees. The cut-off harmonics are generated in pulses with a duration of 0.5 oc.

To introduce the concept of time evolution of the spectrum, we resort to a Morlet wavelet analysis. The Morlet wavelet is defined as:12
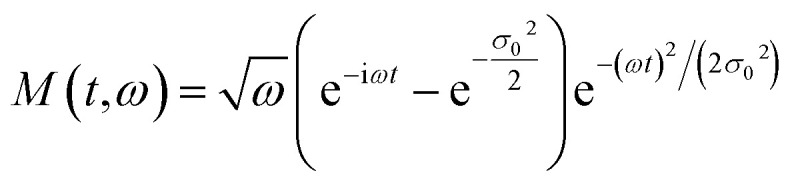
where *σ*_0_ is a parameter that indicates the time-frequency resolution of the integration.^34^ The Morlet wavelet transform of the acceleration is defined as13
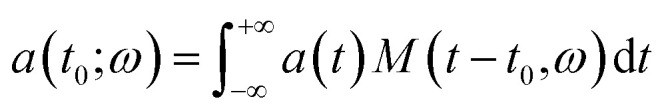
that must replace *a*(*ω*) in the Larmor formula (9). The wavelet transform is a generalization of the Fourier transform; loosely it can be seen as the spectrum of the signal *a* through a temporal window of width *σ*_0_/*ω* and centered at time *t*_0_; by letting *t*_0_ span over the time range of the signal at a fixed *ω*, the temporal evolution of the intensity of the line can be obtained. In our calculation, we choose *σ*_0_ = 2, meaning that within the Morlet wavelet shape there are two oscillations at frequency *ω*.^35^ In Fig. 2 we show the spectra as a function of time for *ϕ* = π − 1/3 and *ϕ* = π/2 for different values of *β*. From this figure, we can see that the harmonics are generated in pulses of duration with half an optical cycle. This feature is understandable by considering that the laser reaches its maximum field strength twice per optical cycle. By changing the *β* parameter and the phase *ϕ* we can control the intensity of the pulses.

The control can be exploited to implement Boolean algebra by assigning the 0 and 1 values to low and high pulse intensities to create a logic gate. As an example, let us suppose that we sample the intensity of the 50th harmonic (in Fig. 2) within the three time intervals centered at 1 oc, 2 oc and 3 oc by means of a sensor. We define four states:

• The state |0〉 when the laser is off and the signal is off

• The state |1〉 when there is only one emission

• The state |2〉 when there are two emissions

• The state |3〉 when we have three emissions

**Fig. 3:**
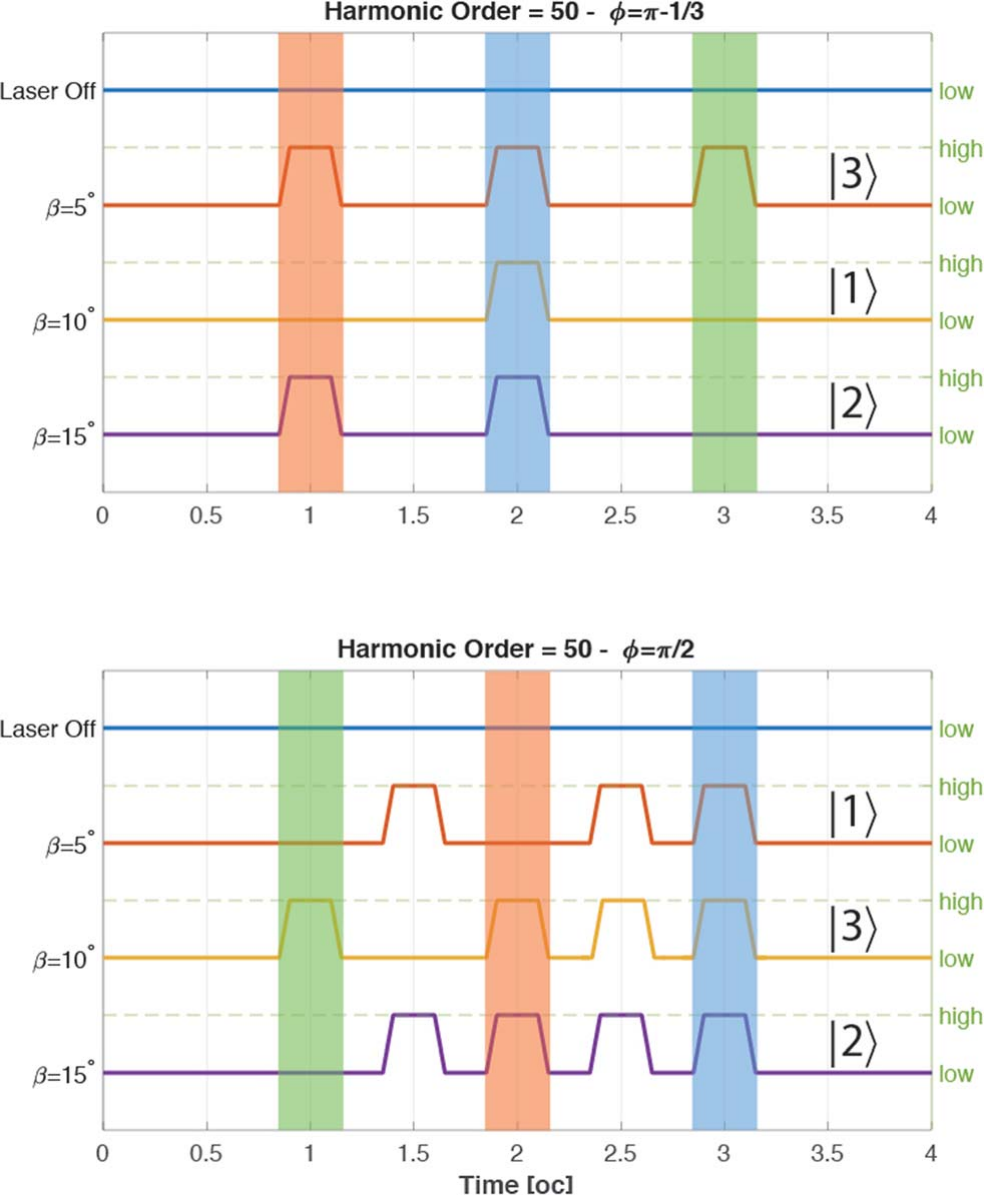
Logic states as a function of time. The presence or the absence of a harmonic emission corresponds to a high or low logic state.

The previous four states are defined independently from the actual time channel of emission of the harmonic: only the number of pulses is relevant. In the top image of Fig. 3 we show the emission of the 50th harmonic *versus* time for different values of *β* with *ϕ* = π − 1/3. Clearly, by setting the value of *β* we can generate one of the four listed states. The presence of the four states permits the creation of ternary and quaternary logic algebras. These algebras have the advantage of being able to perform complex computations with less clock cycles than those in binary algebra. By changing the phase *ϕ* we can modify the pulse emissions and then create new logic states. For example by changing the phase from *ϕ* = π − 1/3 to *ϕ* = π/2 we obtain new states: |1〉 instead of |3〉, |3〉 instead of |1〉, and |2〉 instead of |1〉 (Fig. 3 – bottom). It is of course possible to extend the previous four state table by also taking into account the time channel of emission of the pulse. Indeed, using one sensor tuned at three different times, it is possible to record up to 2^3^ bits, 000 → 111. After lengthy examination of the output of our simulation in Table 1 we list a possible implementation of a complete 8 bit table; of course this requires careful planning of the experiment.

**Table 1:** Implementation of the octal system by changing the CEP *ϕ*. The table has been obtained by keeping *β* = 15°

*ϕ*	1 oc	2 oc	3 oc	Base-8
Laser off	0	0	0	0
0.35	0	0	1	1
0	0	1	0	2
π/2	0	1	1	3
1.06	1	0	0	4
1.46	1	0	1	5
π − 1/3	1	1	0	6
1.16	1	1	1	7

**Fig. 4:**
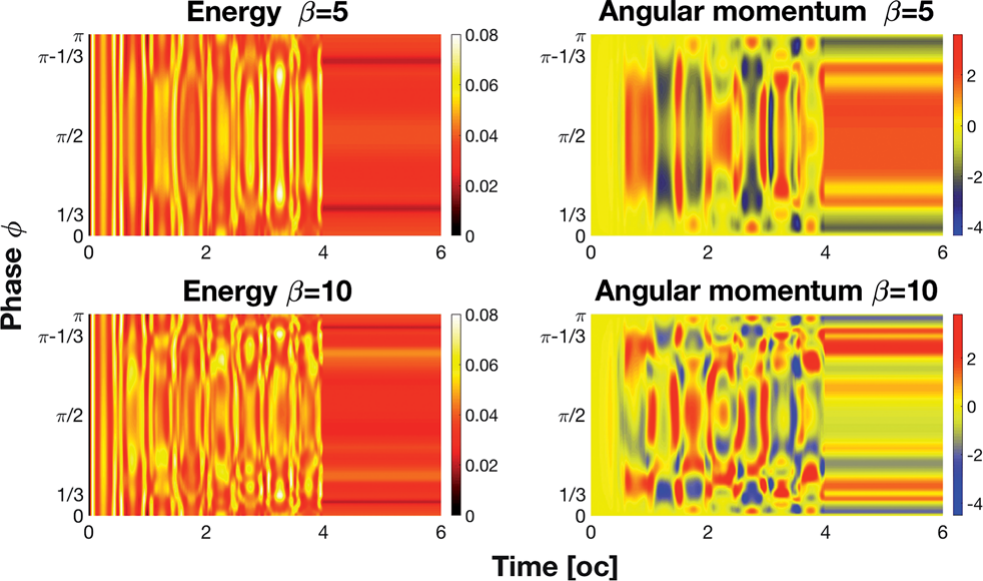
Energy and angular momentum in au absorbed by the electron. Angular momentum variations generate cut-off harmonics.

The final step is the study of the dynamic of the electron to explain the presence of these pulses in the previous wavelet spectrum. We calculated the energy and the angular momentum of the electron for different values of *ϕ*. In Fig. 4 we show the energy and the angular momentum acquired by the electron as a function of time. For *β* = 5° we see that the electron acquires a residual energy centered at *ϕ* = π/2 that corresponds to a residual angular momentum. But if we study the cases with *β* = 10°, we see the final energy and angular momentum moving towards *ϕ* = 1/3 and π − 1/3.

Comparing this figure with Fig. 2, we observe that the harmonic emission occurs when we have an angular momentum inversion that corresponds to a high electron acceleration. After these emissions the electron stores a residual angular momentum.

## Conclusions

In conclusion we have demonstrated that the harmonic generation from a QR depends on the CEP. In particular, we have shown that the system can efficiently emit harmonics if the intensity strength of the electric field polarized along *y* is at most 1/3 of the strength of the *x* polarized component. When the laser is circularly polarized, the electron tends to have a constant speed along the QR. If the polarization of the laser becomes elliptical, the electron will no longer have a constant speed and its acceleration will cause it to emit radiation. In fact, we have a harmonic emission when the electron has strong acceleration. This picture occurs when the laser ellipticity is almost 0.3, which corresponds to ℰ_0*y*_ = ℰ_0*x*_/3. The ellipticity *ε* of the system is:14
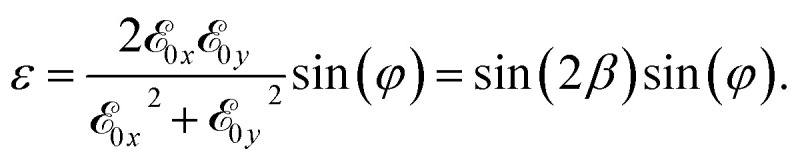


**Fig. 5:**
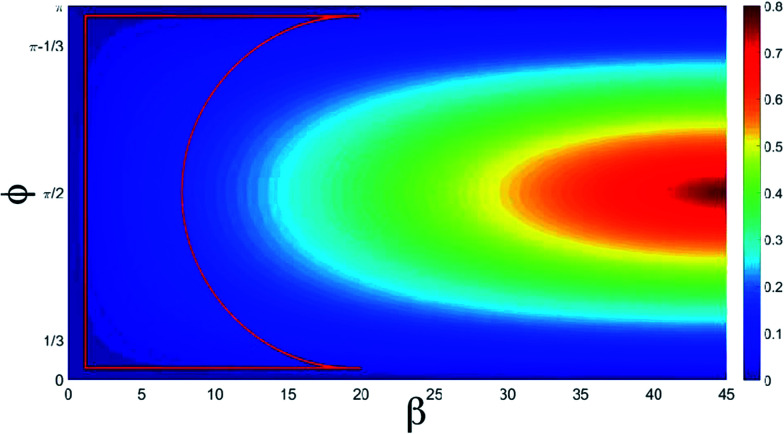
Ellipticity as a function of *β* and *φ* in degrees. The red box represents the area where we have the CEP dependence.

We notice that the ellipticity depends upon *β* and *φ*. When the ellipticity is equal to 1, the laser is circularly polarized, if instead it is equal to 0, the laser is linearly polarized.^36^ To help with the determination of the range of laser parameters that induce a strong acceleration of the electron, in Fig. 5 we show the ellipticity of the whole laser field as a function of *β* and *φ*. We have a CEP dependence for all values within the red box. In this region, we can control the cut-off extension of the spectra by changing the *β* parameter and the phase *φ*. We also notice that in the cut-off region, the harmonics are generated by high angular momentum variations of the electron that correspond to the large electron acceleration mentioned above. By observing the energy and the angular momentum absorbed by the electron, we can see that the electron has several main angular momentum variations that generate the pulses in Fig. 2.

The asymptotic behavior of the last harmonics *versus* the intensity of the *y* component of the laser suggests the possibility of controlling the harmonic emission by changing either the *β* parameter or the phase. In ref. 37 it has been shown that a QR driven by two laser fields can be used to make logic circuits to create gates for logic operations; using the results of this work, the use of QRs to fabricate qubit, qutrit, ququads and an octal system by changing the CEP of the laser is possible with a considerable improvement of the performance of the system.

## Conflicts of interest

There are no conflicts to declare.

## Supplementary Material
